# Thermal Transport in Soft PAAm Hydrogels

**DOI:** 10.3390/polym9120688

**Published:** 2017-12-08

**Authors:** Ni Tang, Zhan Peng, Rulei Guo, Meng An, Xiandong Chen, Xiaobo Li, Nuo Yang, Jianfeng Zang

**Affiliations:** 1 School of Optical and Electronic Information, Huazhong University of Science and Technology, 1037 Luoyu Rd., Wuhan 430074, China; minnie_tang@hust.edu.cn; 2 Key Laboratory of Coal Combustion, Huazhong University of Science and Technology, 1037 Luoyu Rd., Wuhan 430074, China; pengzhanhust@163.com (Z.P.); guorulei@hust.edu.cn (R.G.); anmeng@hust.edu.cn (M.A.); chenxiandong@hust.edu.cn (X.C.); xbli35@hust.edu.cn (X.L.); 3 Nano Interface Center for Energy, School of Energy and Power Engineering, Huazhong University of Science and Technology, 1037 Luoyu Rd., Wuhan 430074, China; 4 Innovation Institute, Huazhong University of Science and Technology, 1037 Luoyu Rd., Wuhan 430074, China

**Keywords:** hydrogel, thermal conductivity, 3ω method, molecular dynamics

## Abstract

As the interface between human and machine becomes blurred, hydrogel incorporated electronics and devices have emerged to be a new class of flexible/stretchable electronic and ionic devices due to their extraordinary properties, such as softness, mechanically robustness, and biocompatibility. However, heat dissipation in these devices could be a critical issue and remains unexplored. Here, we report the experimental measurements and equilibrium molecular dynamics simulations of thermal conduction in polyacrylamide (PAAm) hydrogels. The thermal conductivity of PAAm hydrogels can be modulated by both the effective crosslinking density and water content in hydrogels. The effective crosslinking density dependent thermal conductivity in hydrogels varies from 0.33 to 0.51 Wm^−1^K^−1^, giving a 54% enhancement. We attribute the crosslinking effect to the competition between the increased conduction pathways and the enhanced phonon scattering effect. Moreover, water content can act as filler in polymers which leads to nearly 40% enhancement in thermal conductivity in PAAm hydrogels with water content vary from 23 to 88 wt %. Furthermore, we find the thermal conductivity of PAAm hydrogel is insensitive to temperature in the range of 25–40 °C. Our study offers fundamental understanding of thermal transport in soft materials and provides design guidance for hydrogel-based devices.

## 1. Introduction

Soft hydrogels, such as living tissues and cartilages, are one of the main components of human bodies. Hydrogels are regarded as one big molecule on macroscale with cross-linked polymer network containing significant amounts of water (70–99 wt %) [1]. The intrinsic nature of hydrogels in between a solid and a liquid brings broad applications such as tissue engineering [2], cell encapsulation [3], drug delivery [4], and soft actuators [1]. In addition, the emerging hydrogel-based applications are focused on soft electronics, soft robotics, and machines [5]. Most applications involving grasping, movement, and swelling, thus numerous studies have been carried out to enhance their mechanical properties, such as stretchability, young’s modulus, and fracture energy [6,7,8,9,10].

Besides mechanical properties, heat dissipation in hydrogel-based electronic devices, as well as soft electronics, however, remains largely unexplored, which may be arisen from the fact that hydrogels used in above domains usually fulfill simple actions based on environmental sensitivity without much participating of electronic components. More inspiring examples include hydrogel-based ionic touch panel [11], the steerable smart catheter tip realized by flexible hydrogel actuator [12], double-networking hydrogel-based optical fibers for strain sensors [13], and so on. The attempt on the integration of soft hydrogels with rigid energy consuming components, such as conductive wires, rigid electronic devices, and other functional components, shows prominent potential to revolutionize flexible electronics, soft robotics, and even medical technologies. The possible heat dissipation at the hydrogel-machine interface with encapsulated electronic components and biomedical devices could be a serious issue and needs a better understanding [14,15,16,17].

Existing thermal studies involving hydrogels mainly focused on their nanocomposites [18,19,20] and their thermal stabilities [21,22,23,24] and thermal diffusivity [25]. The hydrogel nanocomposites incorporated with boron nitride (BN) nanopalettes shows an enhanced thermo-responsive time in poly(*N*-isopropyl-acrylamide) (PNIPAm)/BN hydrogels or a better performance as thermal interface materials in poly(acrylic acid) (PAA)/BN hydrogels [26,27]. These investigations pay attention to the role of nanofillers. András Tél et al. investigated the effects of polymer network and the latent heat on heat diffusivity of the PNIPAm hydrogels [17]. While the study of the heat conduction in soft hydrogels is in its infancy stage. Systematic research and an in-depth understanding of the heat transport mechanism in hydrogels are required.

Here, we report the experimental investigation of thermal conduction in hydrogels. The polyacrylamide (PAAm) hydrogel is chosen as a model hydrogel since PAAm is popular for soft electronics and ionic conductors. Both the experimental and simulation results show that the thermal conductivity have an obvious dependence on crosslinker concentrations, which is regarded as a key factor for hydrogels’ mechanical properties [10]. The resultant thermal conductivities of PAAm are in a range of 0.33 ± 0.06~0.51 ± 0.03 Wm^−1^K^−1^, which is slightly below the thermal conductivity of pure water (~0.6 Wm^−1^K^−1^) [28]. We explore the thermal conduction mechanism in hydrogels through equilibrium swelling ratio measurements, scanning electron microscope (SEM) characterization, and equilibrium molecular dynamic (EMD) simulation. The thermal stability of hydrogel with its water content and environment temperature effects has also been evaluated. Our study, on the thermal conduction behavior in hydrogels, may potentially impact both the traditional biomedical field as well as emerging soft electronics. 

## 2. Materials and Methods

### 2.1. Materials Synthesis

**Figure 1:**
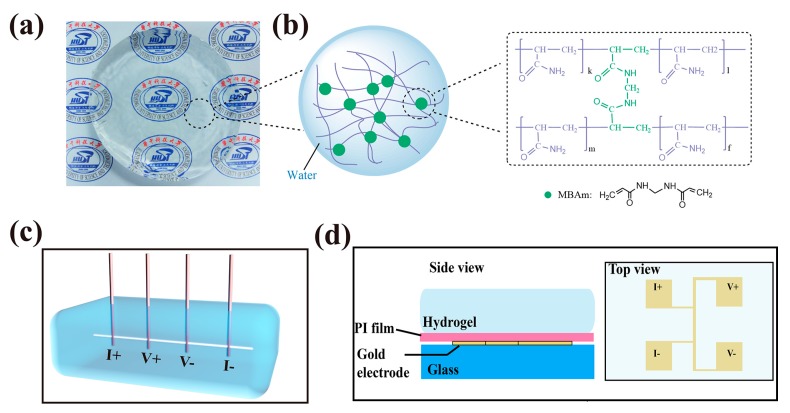
PAAm hydrogel and its experimental setup for thermal conductivity measurement. (**a**) Optical image of the as-prepared PAAm hydrogel sample; (**b**) Schematic of the polymer network with covalent crosslinking through MBAm (green circles) and the molecular structures of the covalently crosslinked polymer chains; (**c**) Schematic of 3ω method setup for thermal conductivity measurement of hydrogels. A platinum wire is deeply immersed in hydrogels and wired out with four copper probes for applied current and voltage measurements. The four brown rods are copper probes and the white line in hydrogel is the Pt wire; (**d**) Schematic of 3ω method for thermal conductivity measurement of hydrogels at different water contents and temperatures. The four Au squares (2 mm in length) on a 1 mm thick glass substrate serve as both the heater and thermometer.

All the following reagents were purchased from Sinopharm Chemical Reagent Co., Ltd. (Shanghai, China) and used without further modified. As prepared PAAm hydrogels are clear and transparent as shown in Figure 1a, PAAm are made from acrylamide (AAm) monomers consisting of carbon double bond and the –CONH_2_ group, which could be co-polymerized by crosslinkers, e.g., *N*-methylenebisacrylamide (MBAm), as shown in Figure 1b.

Samples were synthesized by standard free radicals copolymerization method [7]. AAm powders were dissolved in deionized water (12 wt %) and mixed with different molar ratio of MBAm as a cross-linker. Ammonium persulfate (APS) in water (3 wt %) as initiator and *N*,*N*,*N*′,*N*′-tetramethylethylenediamine (TEMED) as the crosslinking accelerator were added into the above solution in sequence. The gel was then sealed under humid condition for future use.

### 2.2. 3ω Method

When an AC electrical current at angular frequency 1ω is applied at two electrodes, a small voltage signal across the heater to another two electrodes could be detected. The voltage at a frequency of 3ω carrying the thermal effect signal is selected. Combining the relationship among the frequency, voltage and temperature enhancement, the thermal properties of hydrogels can be extracted. Thus, this class of measurement is aptly known as “3ω” method. The thermal conductivity deduced from the 3ω voltage and frequency as follows:

(1) $$\kappa =\frac{\alpha V_{1\omega }^{3}}{8\pi \iota R}\;\frac{1}{dV_{3\omega }/d\ln\omega }$$

where *κ* is the thermal conductivity of the sample, *α* is the temperature coefficient of the heater. For Pt wire in our experiment, *α* is 0.00354 K^−1^. *V*_3ω_ is the 3ω voltage of the heater, *l* is the length of the heater, and *R* is the resistance of the heater before heated.

Figure 1c presents the schematic diagram of our experimental setup of 3ω method for the crosslinker concentration dependent thermal conductivity measurement of the hydrogels. A platinum wire with a diameter of 1 mm connected by four copper electrode rods is immersed in hydrogels. The Pt wire serves as both heater and thermometer. The photo of the real setup and an example for detailed data analysis to obtain the thermal conductivity is shown in Figures S1 and S2 (Supplementary Materials) accordingly.

Figure 1d presents the schematic of 3ω method for thermal conductivity measurement of hydrogels at different water contents and temperatures. Hydrogel sample is put on the patterned Au electrodes, with two electrodes for current input and two for voltage detection. A thin polyethylene (PE) film between the glass slide and sample serves as insulator layer to protect hydrogels. We use a different method because the platinum wire used in Figure 1c is fragile and easily broken by the volume changes in hydrogels. More details on the description of 3ω method can be found in the Sections 1 and 2 in Supplementary Materials.

The feasibility of our measurement setup is validated with test of deionized water at room temperature. The measured thermal conductivity of deionized water is 0.60 Wm^−1^K^−1^, which shows a good agreement with the data in NIST database REFPROP [28]. Multiple measurements were conducted on every hydrogel sample to ensure the reliability and reproducibility of our results.

### 2.3. Equilibrium Swelling Ratio Measurement

For the equilibrium swelling measurements, PAAm gels with different crosslinker concentrations were cut into the same cylinder shape (1 cm in diameter, 3 cm in length). Then, we dry these samples to constant weight in a vacuum oven. To obtain the equilibrium swelling ratio, dry samples were soaked in deionized (DI) water till they reached maximum weight. The weights of the samples are recorded. The equilibrium volumetric swelling ratio *Q* was calculated by Equation (2):

(2) $$Q=\frac{V_{1}+V_{2}}{V_{2}}=\frac{\frac{W_{1}}{\rho_{1}}+\frac{W_{2}}{\rho_{2}}}{\frac{W_{2}}{\rho_{2}}}$$

where *V*_1_ and *V*_2_ are the volume of water fraction after equilibrium swelling and gel after drying, $\rho_{1}$ and $\rho_{2}$ represent the density of water fraction after equilibrium swelling and the polymer, respectively. More details about the theoretical analysis of swelling ratio measurement can be found in the Section 3 in Supplementary Materials.

### 2.4. EMD Simulation

Atomistic simulations provide a powerful tool for validation and interpretation of experimental results [29]. We have performed EMD simulations of thermal transport in hydrogels with different crosslinking concentrations, the mole ratio of crosslinkers to monomers. In simulations, to simplify the case, we ignore water effect in hydrogels and only consider PAAm and MBAm. The crosslinking concentrations (MBAm) are set from 0.0017 to 0.17 mol % at 300 K. All the simulations are carried out utilizing the LAMMPS software package [30]. CVFF [31] was used to calculate bonding and non-bonding interactions. The force-field distribution has accurately predicted thermodynamic properties of components in our system [32]. The details of MD simulations can be found in the Sections 4 and 5 of Supplementary Materials.

### 2.5. Scanning Electron Microscope (SEM)

The PAAm hydrogels with different crosslinker concentration were freeze-dried to keep their original microstructures before analyzed by SEM (FEI, Helios, Waltham, MA, USA) with an accelerating voltage at 2.0 kV.

## 3. Results and Discussion

Various technologies have been developed to measure thermo-physical properties of materials in different states, such as laser-flash method, 3ω method, steady-state method, etc. When it comes to hydrogel samples, which are unique for their gel state, it is challenging and special consideration is needed. The laser-flash method is a simple one and has been reported to measure thermal conductivity of hydrogel nanocomposites with BN nanosheets [26]. But the laser-flash method usually requires opaque samples and thus is not appropriate for the completely transparent samples. For samples with relatively low thermal conductivity, the steady-state technique is not a very accurate method because a large fraction of heat radiated away from the heat flow through the sample during measurement [33]. Another concern for the steady-state method for water-containing hydrogels is the high water evaporation introduced by temperature gradient. While the 3ω method overcomes these problems because the effective thickness of the sample is rather small, in the order of 100 μm, when compared to the typical hydrogel sample size of 1 cm. Thus, the 3ω method is chosen here, the 3ω technique has succeeded in measuring samples in liquid [34,35], in addition to bulk [36], and thin film samples [33].

### 3.1. The Crosslinking Effect

**Figure 2:**
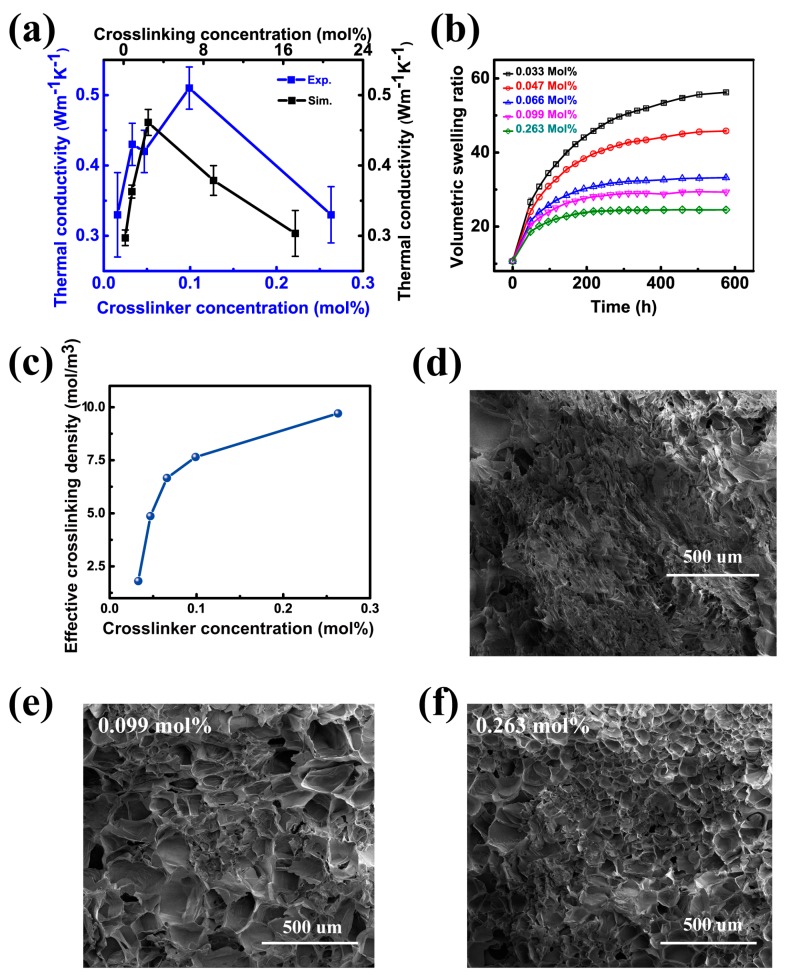
Crosslinking effect of PAAm hydrogels. (**a**) Experimental and simulation results on the thermal conductivity of PAAm hydrogels with different crosslinker concentrations. The blue curves are the experimentally measured thermal conductivities by the 3ω method, and the black curves are the simulated thermal conductivities by molecular dynamics simulations; (**b**) The volumetric swelling ratio of PAAm hydrogels as a function of swelling time; (**c**) The relationship of the effective crosslinking density, and the corresponding crosslinker concentration. SEM images of PAAm gels of constant monomer concentrations and different stages of cross-linking; (**d**) 0.033 mol %; (**e**) 0.099 mol %; (**f**) 0.263 mol %.

The thermal conductivities of PAAm hydrogels as a function of crosslinker concentrations measured by the 3ω method are shown in the blue curves in Figure 2a. The crosslinker concentration is defined as the mole ratio of added crosslinkers to monomers. At the low crosslinker concentration range of 0.016 to 0.099 mol %, the thermal conductivity of hydrogels increases fast and almost linearly from 0.33 ± 0.06 to 0.51 ± 0.03 Wm^−1^K^−1^, giving a 54% enhancement. With the crosslinker concentration further increases to 0.263 mol %, the thermal conductivity decreases to 0.33 ± 0.04 Wm^−1^K^−1^ All the thermal conductivities of hydrogels measured in our experiments are below 0.60 Wm^−1^K^−1^. This means, the thermal conductivity of hydrogels consisting of crosslinked polymer network with significant amount of water seems always below that of pure water. We assume that the intrinsic thermal behaviors of hydrogels are tightly related to their effective crosslinking density and polymer backbones, which is further validated by equilibrium swelling experiment and SEM characterization. The effective crosslinking density in swelling ratio measurement represents the real amount of crosslinkers effectively bonded to polymer chains per unit volume.

To obtain the effective crosslinking density in PAAm hydrogels and understand the corresponding thermal transport behaviors, we implemented the equilibrium swelling experiment first. The water is imbibed into the polymer network to stretch the polymer chains when the hydrogels are soaked in water. The swelling behavior of hydrogels is a typical characteristic of polymers possessing network structures. Flory and Rehner interpreted the swelling behavior with entropy theory: as more and more solvent is absorbed (dissolved) by the polymer, the network is progressively expanded. There exist two opposite entropies, that is, the entropy of chain configuration and the osmotic or mixing entropy. Equilibrium swelling can be attained when these opposite entropies become equal in magnitude. The Flory-Rehner equation is given as:

(3) $$-\left[\ln\left(1-\nu_{p}\right)+\nu_{p}+\chi \nu_{p}^{2}\right]=NV_{s}\left[\nu_{p}^{1/3}-\frac{\nu_{p}}{2}\right]$$

where $v_{p}=Q^{-1}$, $v_{p}$ is the volume swelling ratio, *N* is the effective crosslinking density (mol/m^3^), *V_s_* is the molar volume of the DI water, which is 1.8 × 10^−5^ m^3^/mol. *χ* is the interaction parameter, which is 0.48 [37]. 

According to Equation (3) on effective crosslinking density, Figure 2b presents the time dependent volume swelling ratio of PAAm hydrogels with different crosslinker concentrations. The weight of the hydrogels increases fast at first and reaches saturation when hydrogels are in their equilibrium state after a few days’ swelling. As shown in Figure 2c, the corresponding calculated effective crosslinking density increases fast in the crosslinker concentrations range of 0.016 to 0.099 mol %, and increases slowly when the concentration increases to 0.263 mol %. This result indicates that adding more MBAm into PAAm hydrogels tend to achieve higher effective crosslinking degree in our work. Detailed theoretical analysis of this measurement is included in Supplementary Materials. 

As shown in Figure 2d–f, SEM images of the freeze-dried PAAm hydrogel samples reveal that gels with lower crosslinker concentration (0.033 mol %) exhibit discrete leaflet structures, indicating a low effective crosslinking degree of the polymer network. The ordered honeycomb-like structure was found in PAAm hydrogels with higher cross-linker (0.099 mol % and 0.263 mol %), showing a significantly enhanced effective crosslinking degree. The decreased pore size in the honeycomb structure (Figure 2f) implies a more compact structure as a result of higher degree of effective crosslinking density.

In amorphous polymer, previous studies have revealed that the effective crosslinking density can basically affect thermal conductivity in two ways [38,39]. On one hand, the addition of covalent crosslinking bonds increases thermal conduction pathways between prior non-bonded chain segments, which is verified by the SEM images in Figure 2d–f. On the other hand, more crosslinking bonds can introduce more phonon scattering along the backbone chains, which reduces the phonon mean free path. Particularly, when the effective crosslinking density is low (0~10 mol %), the two effects cancel each other [38,39]; when the effective crosslinking density is large (10~80 mol %), the increase of thermal conduction pathway dominates, which poses a linear increase in thermal conductivity. In our case, the effective crosslinking density is very small in the range of 0~0.263 mol %, two competitive mechanisms occur in this range, which is consistent with previous studies. When the effective crosslinking density is between 0.016 and 0.099 mol %, the enhancement of thermal conductivity could be attributed to the transition from weak van der Waals interactions to strong covalently bonding interactions, which multiplies the thermal transport pathways. While crosslinking network grows (crosslinker concentration 0.099~0.263 mol %), the amount of side chain increases as well which brings in more phonons scattering effect. 

In order to quantitatively study the thermal conductivity of covalently crosslinked PAAm, EMD simulations based on the Green-Kubo method are performed. Simulation details and the theory are shown in Figure S4 (Supplementary Materials). A similar trend of descending after rising in thermal conductivity with crosslinking concentration is observed in our EMD simulation as shown in the black curves in Figure 2a. As we can see, the EMD simulated thermal conductivity is slightly lower than that of those experimentally measured. The difference is enlarged for the sample with relatively higher effective crosslinking density. The polymer chains in experiments are usually much more complicated and longer than polymer chains in ideal EMD simulations. The contribution from the bonded part in longer chains is relatively larger than the shorter chains, which elevate the thermal conductivities in experiments [39]. The addition of greater amounts of crosslinker bonded with chain backbones provides more paths for transportation of phonon. The contribution from larger effective crosslinking density increases as well. In short, we owe the difference between experiment and simulation to the cooperative effect resulting from better thermal conduction of bonded part in longer polymer chains and systems with larger effective crosslinking density. 

### 3.2. The Effect of Water Content

**Figure 3:**
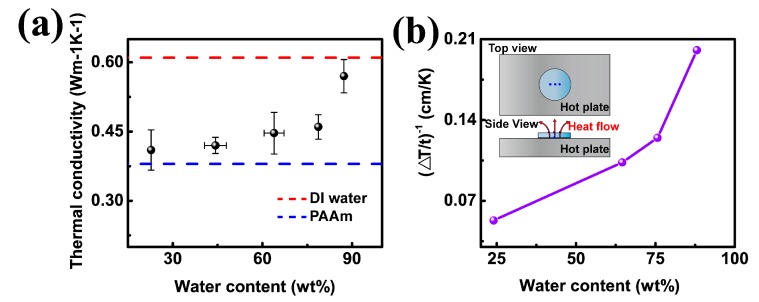
Demonstration of the thermal conductivity of the PAAm hydrogel with 0.099 mol % crosslinker and different water content. (**a**) 3ω based measurement for thermal conductivity of PAAm hydrogels with varied water content; (**b**) Heat dissipation ability with change of water content. Inset: Illustration of measurement of heat dissipation for PAAm hydrogels. The blue dots represent thermocouples. Three thermocouples are put on the surface of sample with distance of about 2 mm between each other, and another one is under the bottom of hydrogel. Temperature gradient come from the difference between the value of the bottom thermocouple and the average value of the three top thermocouples in the equilibrium state.

In EMD simulation, amorphous PAAm chains crosslinked by MBAm are modeled without considering water effect. Actually, the sufficient water content in hydrogels may contribute significantly to the thermal conduction behaviors of the hydrogel samples, which can be inferred from the fact that our EMD simulated results are smaller than the experimental ones. Therefore, we further investigate the thermal conduction behaviors of PAAm hydrogels as a function of water content. To change the water content in PAAm hydrogel, while preserving the original conformation at the same time, ethanol solution is used to dry the hydrogels to obtain samples with different water fractions. As shown in Figure 3a, the thermal conductivity increases gradually from 0.41 ± 0.04 to 0.46 ± 0.03 Wm^−1^K^−1^ as the water fraction increase from 23 to 79 wt %. The thermal conductivity of hydrogel containing 88 wt % water reaches 0.57 ± 0.04 Wm^−1^K^−1^, which is a little bit below that of pure water (0.60 Wm^−1^K^−1^). With water permeated into the polymer backbone, polymer chains in hydrogel could be stretched. In polymer system, stretching is an effective method to enlarge thermal conductive property in polymer system because of the accompanied better chain alignment and less defects [18,31,32]. The thermal conductivity of the drawn nanofibers could be as high as 104 Wm^−1^K^−1^ [40]. It’s interesting if we treat hydrogels as pure water containing cross-linked polymer network. As the fraction of polymer network increases from 0 to 70%, the thermal conductivity keeps decreasing from 0.60 Wm^−1^K^−1^ to a relatively low value, like 0.41 Wm^−1^K^−1^. The addition of the cross-linked polymer network hampers the thermal conduction pathways in pure water. The behind physical mechanism for the thermal transportation in hydrogels involving crosslinking network and water, is complicated, thus requires far more efforts from both experimental and theoretical studies.

The effect of water content on the thermal conductivity of hydrogels is also investigated by measuring heat dissipation curves. Hydrogel samples are cut into a 10 mm thick disk and placed on a 60 °C hot plate at room temperature of 25 °C, as presented in the inset of Figure 3b. The temperature on top surface of the sample is monitored as a function of time by three thermocouples. Heating continues until samples reach their temperature balance state. Figure 3b presents the plot (∆Tt)−1 (∆T is the temperature gradient; *t* is the thickness) as a function of water content, which is in good agreement with Figure 3a. Because the thermal conductivity is inversely proportional to the thickness normalized temperature difference, κ∝(∆Tt)−1. 

### 3.3. The Effect of Temperature

**Figure 4:**
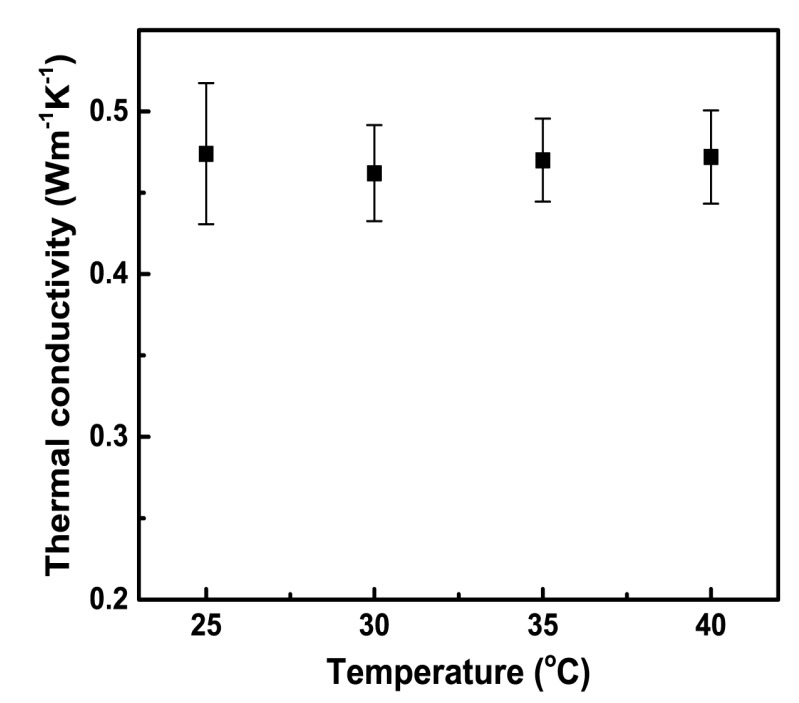
Temperature dependent thermal conductivity of PAAm hydrogels with crosslinker amount of 0.099 mol % measured by the 3ω method.

Since hydrogels are important element for human body, the thermal conduction behaviors of hydrogels at different temperature are potentially significant, for example the human body in a fever state. Thus we evaluate the thermal behavior of PAAm hydrogel in a temperature range of 25~40 °C. We find that hydrogel is insensitive to temperature in this temperature range and exhibits neither deterioration, nor enhancement as shown as Figure 4. 

### 3.4. Temperature Distribution in Hydrogel-Based Electronics

**Figure 5:**
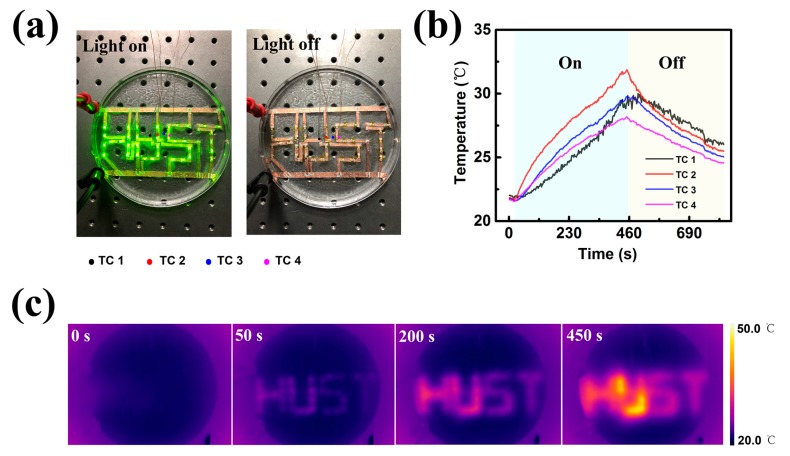
A demonstration of heat dissipation behaviors in the hydrogel-based electronics by K type thermocouples and IR thermal imaging camera. (**a**) Optical image of the electronics with light on and off; (**b**)Time dependent temperature on the surface of the hydrogel (1 cm thick) measured by K type thermocouples; (**c**) IR thermal images of the electronics at different heating time.

Finally, we demonstrate the heat dissipation behaviors of PAAm hydrogel-based electronics with K type thermocouples system (K type series, OMEGA Engineering, Inc., Norwalk, CT, USA and 2700 Multimeter/Data Acquisition System, Keithley Instruments, Inc., Beaverton, OR, USA) and IR thermal imaging camera (IRS-S6, IRS system INC., Shanghai, China). The error of K type thermocouples is ±2.2 °C. With additional error of ±1 °C from the cold junction compensation in the multimeter acquisition system, the comprehensive measurement error of the K type thermocouples system is ±2.5 °C according to the error transfer theory. For the IR thermal imager, the accuracy is in the range of ±2 °C. As shown in Figure 5a, “HUST” (abbreviation of Huazhong University of Science and Technology) patterned LEDs are immersed in a PAAm hydrogel (0.51 ± 0.03 Wm^−1^K^−1^). The LEDs in hydrogels can be turned on and off by adjusting the current in electronics. The thermocouples are placed on the surface of PAAm hydrogel (1 cm thick) and used to detect the temperature distribution in hydrogels encapsulated with the LEDs. As shown in Figure 5b, within 450 s the lighted LED generates a 10 °C increment on the surface of the hydrogel. The resultant heat is accumulated and confined in the surrounding hydrogels of LEDs, as presented in Figure 5b. We further investigate the radial heat dissipation in hydrogel disk with an IR camera in Figure S4 (Supplementary Materials). A 10 °C temperature gradient in a distance of 10 mm within 8 min can be found. Details about the measurement of radial heat dissipation can be found in Section 6 in Supplementary Materials.

The errors involved in temperature measurement using K type thermocouples and IR thermal imager are important and analyzed here. The error of K type thermocouples is ±2.2 °C. With additional error of ±1 °C from the cold junction compensation in the multimeter acquisition system, the comprehensive measurement error of the K type thermocouples system is ±2.5 °C according to the error transfer theory. While the error for the IR thermal imager is in the range of ±2 °C, including random noise and pixel offsets. Although comparable in error, K type thermocouples and IR thermal imager show great difference in accuracy. The accuracy of K type thermocouples is ±0.75%, while that of IR thermal imager is ±2%. Generally, results obtained from K type thermocouples in our experiments are relatively more reliable.

## 4. Conclusions

In summary, the thermal conductivity of PAAm hydrogels has been experimentally measured using the 3ω method. The variation of the thermal conductivity is in a range of 0.33~0.51 Wm^−1^K^−1^ by tuning the effective crosslinking density. The increase of crosslink bonding between the polymer chains favors thermal conduction by increasing conduction pathways at the cost of bringing in more of the phonon scattering effect. The competition mechanism is further supported by the equilibrium swelling ratio measurement, SEM characterization, and molecular simulation analysis. Besides crosslinking effect, thermal conductivity of hydrogel is also dependent on its water fraction. From 23 to 88 wt % of water content, thermal conductivity can be modulated between 0.41 and 0.57 Wm^−1^K^−1^. It’s interesting that over 50% change of thermal conduction of PAAm hydrogel could be achieved only by adjusting its effective crosslinking density and water content, without adding any fillers like nanosheets of graphene or boron nitride. More research efforts regarding the effect of bonding type, polymer chain alignment and porous structure are expected to be devoted in the future to better understand the thermal conduction behaviors in hydrogels. Our study offers an attempt towards understanding of thermal transport in soft materials, and may be beneficial to the design of hydrogel-based devices. 

## Supplementary Materials

The following are available online at www.mdpi.com/2073-4360/9/12/688/s1, Figure S1: Picture of 3ω method experimental setup, Figure S2: Experimental data for sample with 0.033 wt % crosslinker concentration at room temperature 295.1 K, Figure S3: Molecular dynamics simulation cell and one example of data analysis, Figure S4: Illustration of radial heat transportation in hydrogels with a heat source in the center of samples.
