# Supplementary material for: Thermal Transport in Soft PAAm Hydrogels
Source: Polymers (Basel). 2017 Dec 8;9(12):688. doi: 10.3390/polym9120688 (PMC6418834; doi:10.3390/polym9120688)
Supplement: Supplementary file 1 [file polymers-09-00688-s001.pdf]

Supplementary Materials

Thermal transport in soft PAAm hydrogels

Ni Tang[1,†], Zhan Peng[2,3,†], Rulei Guo[2,3,†], Meng An[2,3], Xiandong Chen[2,3], Xiaobo Li[2,3], Nuo Yang[2,3,*] and Jianfeng Zang[1,4,*]

1 School of Optical and Electronic Information, Huazhong University of Science and Technology, 1037 Luoyu Rd., Wuhan 430074, China; Minnie_tang@hust.edu.cn (N.T.)
2 Key Laboratory of Coal Combustion, Huazhong University of Science and Technology, 1037 Luoyu Rd., Wuhan 430074, China; pengzhanhust@163.com (Z.P.); guorulei@hust.edu.cn (R.G.); anmeng@hust.edu.cn (M.A.); chenxiandong@hust.edu.cn (X.C.); xbli35@hust.edu.cn (X.L.)
3 Nano Interface Center for Energy, School of Energy and Power Engineering, Huazhong University of Science and Technology, 1037 Luoyu Rd., Wuhan 430074, China
4 Innovation Institute, Huazhong University of Science and Technology, 1037 Luoyu Rd., Wuhan 430074, China
* Correspondence: nuo@hust.edu.cn (N.Y.); jfzang@hust.edu.cn (J.Z.); Tel.: +86-27-87792461 (J.Z.)
† These authors contributed equally to this work.

Table of Contents

1. 3-ω method for crosslinking density dependent thermal conductivity in hydrogels
   1.1 Theoretical analysis ······P2-3
   1.2 Experimental setup······P3-5
2. 3-ω method for measure of the water content and temperature dependent thermal conductivity in hydrogels······P5-6
3. Equilibrium swelling ratio measurement······P6-7
4. MD simulation details······P7-8
5. Table S1 The potential details······P8-12
6. Thermal distribution in PAAm hydrogels······P12

1. 3-ω method for crosslinking density dependent thermal conductivity in hydrogels

1.1 Theoretical analysis

A sinusoidal current of frequency ω goes through the platinum wire. Then, the fluctuation of the temperature rise (ΔT) is 2ω due to the Joule heating. As the electrical resistance of the platinum wire has a linear relationship with temperature, the fluctuation of the wire resistance is also 2ω. Consequently, the current of frequency ω multiply by the resistance of frequency 2ω produces a small voltage signal of frequency 3ω. This small voltage includes the information of the thermophysical properties of the liquid.

An electrical current of frequency ω and magnitude I_0 is applied to the heater.

$$\mathrm{I}(t) = I_0 \cos(\omega t) \qquad (1)$$

Due to the resistance change of the heater is much smaller than its resistance R_0 at environment temperature, the power dissipated by the heater is

$$\mathrm{P} = I^2 R \approx I_0^2 R_0 \frac{\cos(2\omega t)+1}{2} \qquad (2)$$

For small temperature changes, the resistance of the wire varies linear with temperature as

$$\mathrm{R}(t) = R_0[1 + \alpha \triangle T \cos(2\omega t - \Phi)] \qquad (3)$$

Where α is the temperature coefficient of resistance (TCR),

$$\alpha = \frac{1}{R}\frac{dR}{dT} \qquad (4)$$

The resulting voltage across the wire is obtained through the input current multiplying by the heater resistance.

$$\mathrm{V}(t) = I(t)R(t) = I_0 R_0 \cos(\omega t) + \frac{1}{2} I_0 R_0 \alpha \Delta T \cos(\omega t - \Phi)$$

$$+\frac{1}{2} I_0 R_0 \alpha \Delta T \cos(3\omega t - \Phi) \qquad (5)$$

In such a structure with an AC electrical current passing through the heater, the heat generated and diffused into the sample can be described by the following equation:

$$\frac{\partial \mathrm{T}}{\partial \mathrm{t}} = \alpha \left(\frac{\partial^2 T}{\partial r^2} + \frac{1}{r}\frac{\partial T}{\partial r}\right) \qquad (6)$$

and the initial and boundary conditions is:

$$\mathrm{r} \rightarrow \infty, \mathrm{U}(r) = 0 \qquad (7)$$

$$r = r_0, \frac{\partial T}{\partial r} = -\frac{P}{2\pi r_0 l \kappa} \tag{8}$$

So, solving the equation, we can get

$$\Delta T = \frac{V_{1\omega}^2}{2\pi l R_0 \kappa}\left[-\frac{1}{2}\ln(\omega) + \frac{1}{2}\ln\frac{D}{r^2} + \ln 2 - r - i\frac{\pi}{4}\right] \tag{9}$$

Where $V_{1\omega}$ the voltage of the heater, and R_0 is the resistance of the heater before heated, l is the length of heater, κ is the thermal conductivity of sample, *D* is the thermal diffusivity of sample, and *r* is the radius of heater. From Eq. 5, we can get the temperature rise by measuring the 3ω voltage,

$$\Delta T = \frac{2V_{3\omega}}{V_{1\omega}\alpha} \tag{10}$$

$V_{3\omega}$ is the 3ω voltage of the heater. α is the temperature coefficient of the heater. According to Eq. 9 and 10, we measure the different 3 ω voltage of different frequency, we can get the thermal conductivity. According to the literature[1,2], the thermal conductivity can be deduced from 3ω voltage and frequency as follows:

$$\kappa = \frac{\alpha V_{1\omega}^3}{8\pi \iota R}\frac{1}{dV_{3\omega}/d\ln\omega} \tag{11}$$

where κ is the thermal conductivity of the sample, α is the temperature coefficient of the heater. For Pt wire in our experiment, α is 0.00354 PPm/ºC. $V_{3\omega}$ is the 3ω voltage of the heater, *l* is the length of the heater, and *R* is the resistance of the heater before heated.

1.2 Experimental setup

The diagram of the measurement system is shown in Figure S1. The platinum wire is welded on copper rods. The diameter of the Pt wire is 20 um. An alternating current (AC) from the signal generator passes though the platinum wire and the resistor. The voltage signal of the wire and the resistor are then input into the Lock-in Amplifier though the differential amplifiers AMP03. The resistor is adjusted to balance the 1ω voltage signal of the platinum wire because the 1ω voltage is thousands of times larger than the 3ω voltage[3]. If the 1ω voltage is not eliminated, the obtained 3ω voltage may not be accuracy. The Lock-in Amplifier can get the 3ω voltage signal through the differential input A-B.

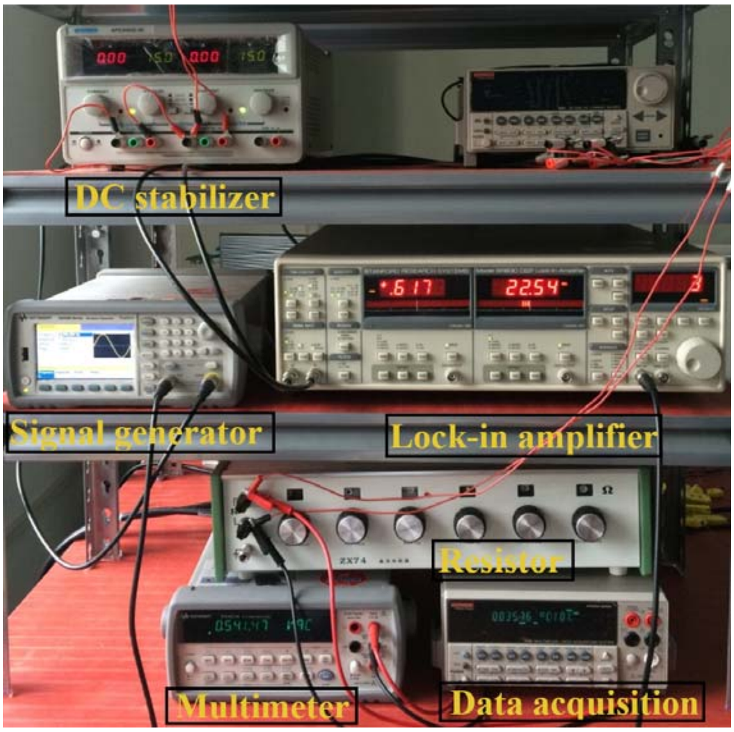

Figure S1. Picture of 3-ω method experimental setup.

According to the relationship between the length of the wire and the penetration depth, the minimum frequency we can estimate to 0.007 Hz. Similarly , the radius of the wire must be much more smaller than the penetration depth[4], we can also estimate the maximum frequency to about 7 Hz. So ,the measured frequency should be included in the range from 0.007 Hz to 7 Hz. Figure S2 exhibits the measured 3ω voltage as a function of Logω for hydrogel with 35 ul concentration at 295.1 K. As the relation of 3ω voltage and Logω is not linear when the frequency is larger than10 Hz, the lower frequency is only used to calculate. The thermal conductivity is 0.44 $Wm^{-1}K^{-1}$. The same procedure is applied to the other samples.

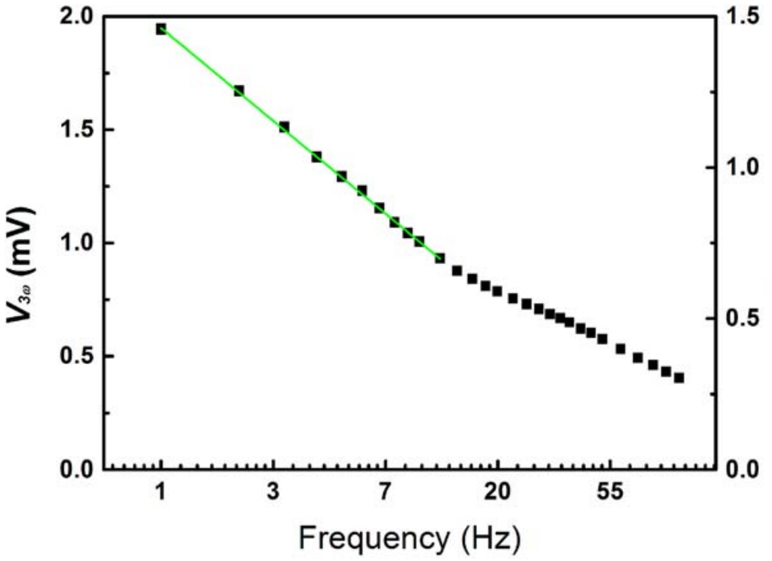

Figure S2. Experimental data for sample with 0.033wt% crosslinker concentration at room temperature 295.1K. The black square is the measured value of 3ω voltage. The green solid line is the linear fitting .At low frequency, $V_{3\omega}$ has a linear relationship with logarithmic frequency.

2. 3-ω method for measure of the water content and temperature dependent thermal conductivity in hydrogels

We use another 3-ω method in which the heat wire isn't put in hydrogels. Similar to the aforementioned, an alternating current (AC) passes through the metal heater at a frequency ω, which heats the sample periodically and generates oscillations in the resistance of the metal line at a frequency 2ω. The oscillating resistance component is coupled with the driving current to generate the 3ω voltage component over the heater. The magnitude and frequency dependence of the 3ω voltage depends on the thermal properties of the glass substrate, insulating layer and the sample of hydrogel. With all the thermal properties of other materials having be known, we can get the thermal properties of hydrogel from experiment data with parameter fitting. This method can be named by the two-direction asymmetric heat transfer model[5,6]. This method has been successfully used to measure the thermal conductivity of porous SiO_2 film[7], copper nanowires array[8] and Metal Inverse Opals[9].

The relationship of the temperature rise of the heater, 3ω voltage and 1ω voltage is that[10],

$$\Delta T(2\omega)=\frac{2V_{3\omega}}{V_{1\omega}\beta} \tag{12}$$

$V_{1\omega}$is the 1ω voltage, $V_{3\omega}$ is the in-phase 3ω voltage, β is the temperature coefficient of the metal wire. The temperature rise of the metal heater is[11]

$$\Delta T(2\omega)=\frac{P}{\pi l}\int_0^{\infty}\frac{1}{2\gamma_2}\frac{(B^{+}+B^{-})(A^{+}+A^{-})}{A^{+}B^{-}-A^{-}B^{+}}\frac{\sin^2(\lambda b)d\lambda}{(\lambda b)^2} \tag{13}$$

Where P is the power of the metal heater, l and b are length and half-width of the heater, respectively, λ is the integral variable in Fourier space, $\gamma_i=\kappa_i\sqrt{\lambda^2+i2\omega/\alpha_i}$, $\alpha_i=\kappa_i/C_i$, C_i is the volume heat capacity of the ith layer. A^+, A^-, B^+ and B^- are dimensionless parameters solved using a recursive matrix method. A^+ and A^- are(A^+ is the top item in the matrix, A^- is the beneath item)

$$\widetilde{A}=\frac{1}{2\gamma_2}\begin{pmatrix}\gamma_2+\gamma_1+\gamma_1\gamma_2 R & \gamma_2-\gamma_1-\gamma_1\gamma_2 R\\ \gamma_2-\gamma_1+\gamma_1\gamma_2 R & \gamma_2+\gamma_1-\gamma_1\gamma_2 R\end{pmatrix}\begin{pmatrix}e^{\mu_1 d} & 0\\ 0 & e^{-\mu_1 d}\end{pmatrix}\frac{1}{2\gamma_1}\begin{pmatrix}\gamma_1+\gamma_0 & \gamma_1-\gamma_0\\ \gamma_1-\gamma_0 & \gamma_1+\gamma_0\end{pmatrix}\begin{pmatrix}1\\0\end{pmatrix} \quad (3)$$

And $\mu_i=\sqrt{\lambda^2+i2\omega/\alpha_i}$ is the penetrate depth in Fourier space[6], R is the interfacial thermal resistance between the metal line and the membrane. d is the thickness of the insulation layer. B^+ and B^- are (B^+ is the top item in the matrix, B^- is the beneath item)

$$\widetilde{B}=\begin{pmatrix}0\\1\end{pmatrix} \quad (14)$$

3. **Equilibrium swelling ratio measurement**

The equilibrium volumetric swelling ratio Q was calculated by equation (2):

$$Q=\frac{V_1+V_2}{V_2}=\frac{\frac{W_1}{\rho_1}+\frac{W_2}{\rho_2}}{\frac{W_2}{\rho_2}}=1+\frac{\rho_2}{\rho_1}\cdot\frac{W_1}{W_2} \quad (15)$$

where V_1 and V_2 are the volume of water fraction after equilibrium swelling and gel after drying, ρ_1 and ρ_2 represent the density of water fraction after equilibrium swelling and the polymer, respectively.

The equilibrium volume fraction of polymer in the hydrogels v_p is:

$$v_p=Q^{-1} \quad (16)$$

Flory-Rehner equation, which is based on the equilibrium swelling of polymer in solvent for three dimensional networks of randomly coiled chains, is suitable for our materials. The Flory-Rehner equation is given as:

$$-\left[\ln(1-v_p)+v_p+\chi v_p^2\right]=NV_s\left[v_p^{1/3}-\frac{v_p}{2}\right] \quad (17)$$

where N is the crosslinking density (mol/m^3), V_s is the molar volume of the DI water, which is $1.8\times10^{-5}m^3/mol$. χ is the interaction parameter, which is 0.48[12].

The average molecular weight $\overline{M_c}$ between crosslinks is given as:

$$-\left[\ln(1-v_p)+v_p+\chi v_p^2\right]=\frac{\rho_2}{\overline{M_c}}V_s\cdot v_p^{1/3} \quad (18)$$

According to the above expressions, the crosslinking density N increase with increasing of equilibrium swelling ratio, and the average molecular weight of a chain between adjacent crosslinking points shows an opposite trend compared with crosslinking density.

4. MD simulation details

The simulation cell and its enlarged view are presented in Figure S4a and S4b. The lattice thermal conductivity of hydrogels is calculated by equilibrium molecular dynamics (EMD) based on the Green-Kubo formula[13] as

$$\kappa = \frac{1}{3\kappa_B T^2 V} = \int_0^{\infty} \langle \vec{J}(\tau) \cdot \vec{J}(0) \rangle \, d\tau \tag{19}$$

where κ is the thermal conductivity, k_B is the Boltzmann constant, V and T is the volume of the simulation cell temperature, respectively. $\vec{J}(\tau) \cdot \vec{J}(0)$ is the heat current autocorrelation function (HCACF). The angular bracket denotes ensemble average. The heat current is given by

$$\vec{J}(\tau) = \sum_i \vec{v}_i \, \varepsilon_i + \frac{1}{2} \sum_{i,j} \vec{r}_{ij} \left(\vec{F}_{ij} \cdot \vec{v}_i \right) \tag{20}$$

where $\vec{v}_i$ and ε_i are the velocity vector and energy (kinetic and potential) of particle i, respectively. $\vec{r}_{ij}$ and $\vec{F}_{ij}$ are the inter-particle separation vector and force vector between particles i and j, respectively. In MD simulation, the temperature T_{MD} is calculated from the kinetic energy of atoms according to the Boltzmann distribution[14]:

$$\langle E \rangle = \sum_{i=1}^{N} \frac{1}{2} m v_i^2 = \frac{1}{2} N \kappa_B T_{MD} \tag{21}$$

All the simulations are carried out utilizing the LAMMPS software package. Consistent Valance Force Field（CVFF）potential was used for bonded as well as non-bonded interactions. The potential details is shown in Table S1. The force-field have successfully predicted accurate thermodynamic properties of interests for our system of interest[15]. Taking the PAAm with crosslinking concentration of 2.459 mol % for example, we obtain the time dependent heat current autocorrelation function (HCACF), as shown in Figure S4c. The thermal conductivity extracted from the HCACF curve is shown in Figure S4d.

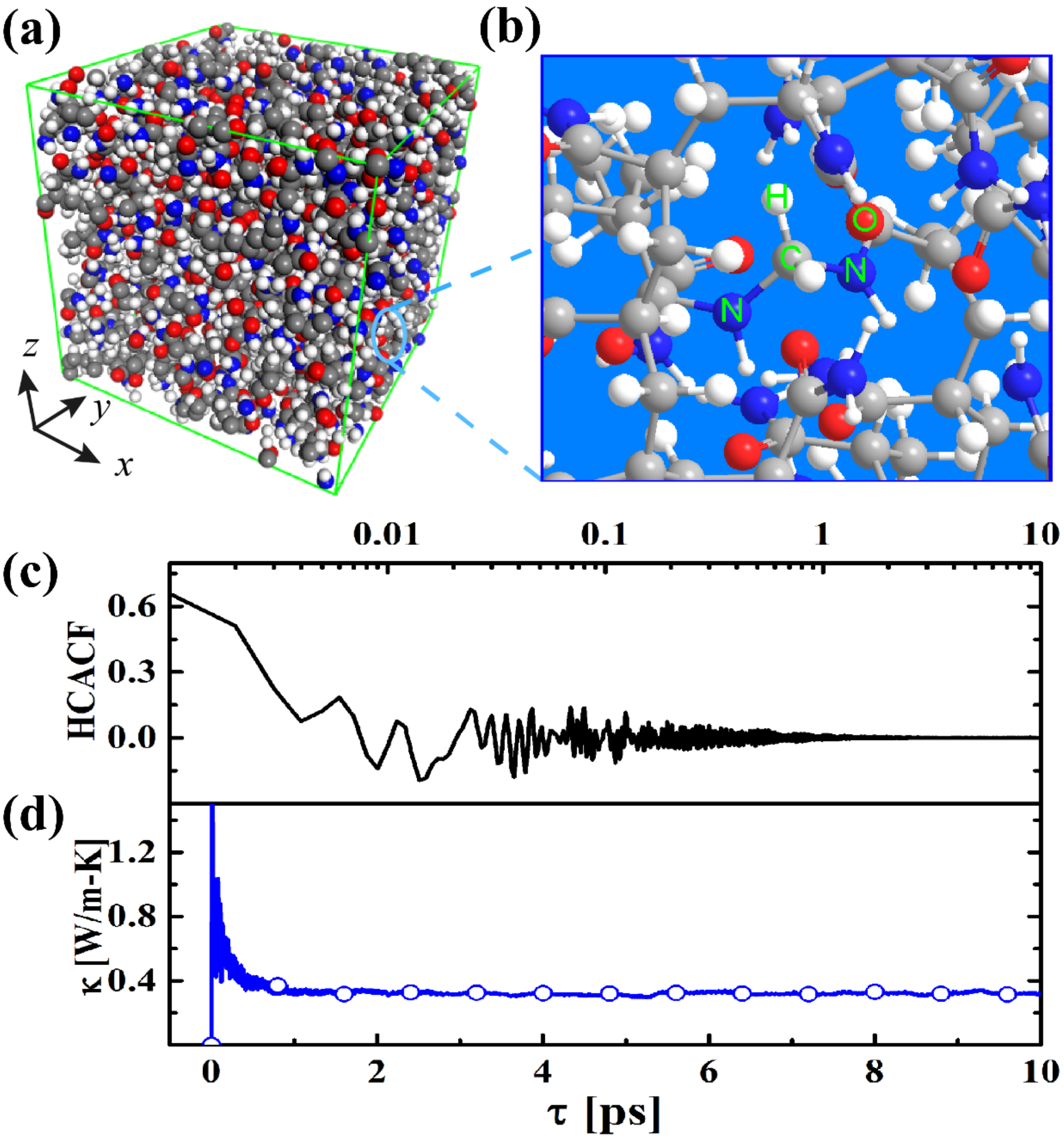

Figure S3. Molecular dynamics simulation cell setup and one example of data analysis. (a) Molecular dynamics simulation cell. The simulation cell size is 3.76 nm×3.76 nm×3.76 nm, and its density is 1.36 g/cm^3. (b) The enlarged view showing the covalently crosslinked polymer chains. The data analysis of PAAm with 2.459 mol% crosslinking density: (c) time dependent HCACF curve. (d) The lattice thermal conductivity obtained from the time integration of HCACF curve.

5. Table S1 The potential details

The velocity Verlet algorithm is employed to integrate equation of motion, and the time step is set as 0.1fs. At the beginning of a simulation for a thermal conductivity prediction, the system is run in the NVT ensemble to set the temperature. After 50 ps, the simulations run in the NPT ensemble for 500 ps to relax the structure. After relaxation, the converged values of both the cell size and the potential energy obtained, which make sure that there is no stress or strain effects. Then the simulation is switched to run in the NVE ensemble, and the HCACF is obtained over 500 ps. The thermal conductivity is then obtained from the integral of the

HCACF (Equation 15).

Table S1 Force field parameters adopted in CVFF for molecular dynamics simulations.

Bond Parameters		
bond	K_r (eV/Å^2)	r_{eq} (Å)
C2–H	14.770	1.105
C2–C1	13.994	1.526
C1–H	14.770	1.105
C1-C	12.276	1.520
C-O	26.682	1.230
C-N2	16.825	1.320
N2-H	19.837	1.026
C=-C=	28.411	1.330
C=-H	15.680	1.090
C2-C=	13.998	1.500
C-C=	13.998	1.500
C-N	16.825	1.320
C2-N	16.373	1.460
N-H	20.964	1.026

Angle Parameters		
Angle	K_θ (eV/Å^2)	θ_{eq} (degrees)
H-C2-H	1.712	106.4
C1-C2-H	1.925	110.0
C1-C2-C1	2.021	110.5
C2-C1-H	1.925	110.0
C2-C1-C2	2.021	110.5
C2-C1-C	2.021	110.5
C-C1-H	1.951	109.5
C1-C-O	2.949	120.0

C1-C-N2	2.320	114.1
N2-C-O	2.949	120.0
C-N2-H	1.626	115.0
H-N2-H	1.431	125.0
C=-C=-H	1.466	121.2
C2-C=-C=	1.570	122.3
C-C=-C=	1.570	122.3
C2-C=-C	2.168	120.0
C=-C2-H	1.925	110.0
O-C-C=	2.168	120.0
N2-C-C=	2.320	114.1
C1-C-N	2.320	114.1
O-C-N	2.949	120.0
C2-N-C	4.813	118.0
C-N-H	1.626	115.0
C2-N-H	1.518	122.0
N-C2-H	2.233	109.5

Van der Waals Parameters

atom type	ε (eV)	σ (Å)
C2	0.0017	3.8754
C1	0.0017	3.8754
C	0.0064	3.6170
N2	0.0073	3.5013
O	0.0099	2.8598
C=	0.0064	3.6170
N	0.0073	3.5012
H	0.0017	2.4450

Dihedral Parameters			
Dihedral type	K (eV)	d	n
H-C2-C1-H	0.00686	1	3
H-C2-C1-C2	0.00686	1	3
H-C2-C1-C	0.00686	1	3
C1-C2-C1-H	0.00686	1	3
C1-C2-C1-C2	0.00686	1	3
C1-C2-C1-C	0.00686	1	3
C1-C-N2-H	0.0651	-1	2
O-C-N2-H	0.0651	-1	2
C=-C2-C1-C2	0.00686	1	3
C=-C2-C1-H	0.00686	1	3
C=-C2-C1-C	0.00686	1	3
C2-C=-C=-H	0.177	-1	2
C-C=-C=-H	0.177	-1	2
C1-C2-C=-C=	0.00915	1	3
H-C2-C=-C=	0.00915	1	3
C1-C2-C=-C	0.00915	1	3
H-C2-C=-C	0.00915	1	3
O-C-C=-C=	0.0195	-1	2
N2-C-C=-C=	0.0195	-1	2
O-C-C=-C2	0.0195	-1	2
N2-C-C=-C2	0.0195	-1	2
N2-C-C=-C2	0.0195	-1	2
C=-C-N2-H	0.0650	-1	2
C1-C-N-C2	0.139	-1	2
C1-C-N-H	0.0520	-1	2
O-C-N-C2	0.165	-1	2

O-C-N-H	0.0781	-1	2

6. Thermal distribution in PAAm hydrogels

We also demonstrate the heat dissipation behaviors of the hydrogel with a heat source inside the sample through IR thermal imaging camera. As shown in Figure 5a and 5b, an electrically heated graphite rod is plugged through the PAAm hydrogel block (0.51 $Wm^{-1}K^{-1}$) to provide a heat source and the thermal camera records the time dependent temperature distribution on the top view of samples. Figure 5c presents the heat propagates radically from the heat source to the outer circles, generating a 10℃temperature gradient in a distance of 10 mm within 8 min.

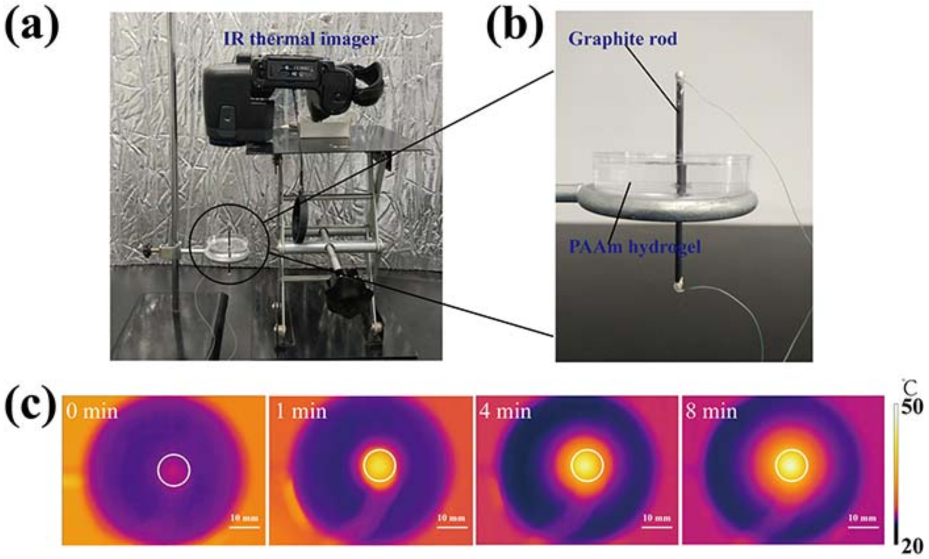

Figure S4. A demonstration of radial heat dissipation behaviors in the hydrogel by IR thermal imaging camera. (a) Optical image of the whole measurement system. The IR thermal camera is fixed on top of the hydrogel sample to record the thermal change. (b) Optical image of the sample. An electrically heated graphite rod is plugged through the PAAm hydrogel sample with crosslinker amount of o.099 mol%. (c) IR thermal images of the hydrogel at different heating time.

Reference

1. Paul, G.; Chopkar, M.; Manna, I.; Das, P. K. Techniques for Measuring the Thermal Conductivity of Nanofluids: A Review. *Renew. Sustain. Energy Rev.* **2010**, *14* (7), 1913–1924.
2. Wang, Z. L.; Tang, D. W.; Liu, S.; Zheng, X. H.; Araki, N. Thermal-Conductivity and Thermal-Diffusivity Measurements of Nanofluids by 3ω Method and Mechanism Analysis of Heat Transport. *Int. J. Thermophys.* **2007**, *28* (4), 1255–1268.
3. Dames, C.; Chen, G. 1omega, 2omega, and 3omega Methods for Measurements of Thermal Properties. *Rev. Sci. Instrum.* **2005,** 76, 1033.

4. Yusibani, E.; Woodfield, P. L.; Fujii, M.; Shinzato, K.; Zhang, X.; Takata, Y. Application of the Three-Omega Method to Measurement of Thermal Conductivity and Thermal Diffusivity of Hydrogen Gas. *Int. J. Thermophys.* **2009**, *30* (2), 397–415.
5. Feldman, A. Algorithm for Solutions of the Thermal Diffusion Equation in Stratified. *High Temp. Press.* **1999,** 31, 293-298.
6. Kim, J. H.; Feldman, A.; Novotny, D. Application of the Three Omega Thermal Conductivity Measurement Method to a Film on a Substrate of Finite Thickness. *J. Appl. Phys.* **1999**, *86* (7), 3959–3963.
7. Hopkins, P.; Kaehr, B.; Phinney, L.; Koehler, T.; Grillet, A.; Dunphy, D.; Garcia, F.; Brinker, C. J. Measuring the Thermal Conductivity of Porous, Transparent SiO2 Films With Time Domain Thermoreflectance. *J. Heat Transfer.* **2011,** 133, 989-955.
8. Barako, M. T.; Roy-Panzer, S.; English, T. S.; Kodama, T.; Asheghi, M.; Kenny, T. W.; Goodson, K. E. Thermal Conduction in Vertically Aligned Copper Nanowire Arrays and Composites. *ACS Appl. Mater. Interfaces.* **2015**, *7* (34), 19251–19259.
9. Barako, M. T.; Sood, A.; Zhang, C.; Wang, J.; Kodama, T.; Asheghi, M.; Zheng, X.; Braun, P. V.; Goodson, K. E. Quasi-Ballistic Electronic Thermal Conduction in Metal Inverse Opals. *Nano Lett.* **2016**, *16* (4), 2754–2761.
10. Cahill, D. G. Thermal Conductivity Measurement from 30 to 750 K: The 3?? Method. *Rev. Sci. Instrum.* **1990**, *61* (2), 802–808.
11. Bauer, M. L.; Norris, P. M. General Bidirectional Thermal Characterization via the 3?? Technique. *Rev. Sci. Instrum.* **2014**, *85* (6), 802.
12. Baysal, B. M.; Kayaman, N. Swelling of Polyacrylamide Gels in Polyacrylamide Solutions. **1997**, 1313–1320.
13. Mi, X. Y.; Yu, X.; Yao, K. L.; Huang, X.; Yang, N.; Lü, J. T. Enhancing the Thermoelectric Figure of Merit by Low-Dimensional Electrical Transport in Phonon-Glass Crystals. *Nano Lett.* **2015**, *15* (8), 5229–5234.
14. Nanolaminates, W. A. O.; Costescu, R. M.; Cahill, D. G.; Fabreguette, F. H.; Sechrist, Z. A.; George, S. M. Ultra-Low Thermal Conductivity. *Science.* **2004**, *303*, 989–991.
15. Plimpton, S. Fast Parallel Algorithms for Short-Range Molecular Dynamics. *Journal of Computational Physics*. **1995,** 117, 1–19.
